# Point Pair Feature-Based Pose Estimation with Multiple Edge Appearance Models (PPF-MEAM) for Robotic Bin Picking

**DOI:** 10.3390/s18082719

**Published:** 2018-08-18

**Authors:** Diyi Liu, Shogo Arai, Jiaqi Miao, Jun Kinugawa, Zhao Wang, Kazuhiro Kosuge

**Affiliations:** 1 Graduate School of Engineering, Tohoku University, Aramaki Aza Aoba 6-6-01, Aoba-Ku, Sendai 980-8579, Japan; arai@irs.mech.tohoku.ac.jp (S.A.); Kinugawa@irs.mech.tohoku.ac.jp (J.K.); 2008wangzhao@gmail.com (Z.W.); kosuge@irs.mech.tohoku.ac.jp (K.K.); 2 Jack Baskin School of Engineering, University of California, Santa Cruz, CA 95064, USA; jmiao3@ucsc.edu

**Keywords:** robotic bin picking, pose estimation, Boundary-to-Boundary-using-Tangent-Line (B2B-TL), Multiple Edge Appearance Models (MEAM)

## Abstract

Automation of the bin picking task with robots entails the key step of pose estimation, which identifies and locates objects so that the robot can pick and manipulate the object in an accurate and reliable way. This paper proposes a novel point pair feature-based descriptor named Boundary-to-Boundary-using-Tangent-Line (B2B-TL) to estimate the pose of industrial parts including some parts whose point clouds lack key details, for example, the point cloud of the ridges of a part. The proposed descriptor utilizes the 3D point cloud data and 2D image data of the scene simultaneously, and the 2D image data could compensate the missing key details of the point cloud. Based on the descriptor B2B-TL, Multiple Edge Appearance Models (MEAM), a method using multiple models to describe the target object, is proposed to increase the recognition rate and reduce the computation time. A novel pipeline of an online computation process is presented to take advantage of B2B-TL and MEAM. Our algorithm is evaluated against synthetic and real scenes and implemented in a bin picking system. The experimental results show that our method is sufficiently accurate for a robot to grasp industrial parts and is fast enough to be used in a real factory environment.

## 1. Introduction

The bin picking task must be automated in order to automate all tasks in factories such as machine loading, assembly and order fulfillment. The bin picking task refers to grasping individual parts from an unordered pile of parts in a carrier or box [1]. To grasp a part, it is first necessary to locate it. Therefore, the pose estimation of distinct parts in a scene is important [2].

Many pose estimation algorithms have been proposed to solve this key problem. They can be divided into three categories according to the input dimensions: 2D (image), 3D (depth image, point cloud) and 2D plus 3D (organized point cloud).

Algorithms with 2D input always use a camera to capture the scene. Collet et al. have proposed a method with a single image as the input. A method of the construction of 3D models based on the structure-from-motion bundle adjustment algorithm, using several images with local descriptors [3], is utilized. While the image of a scene is captured, local descriptors are extracted and matched to the prepared model. A novel combination of the Random Sample Consensus (RANSAC) and mean shift algorithms is used to register multiple instances of each object. However, this method is incapable of detecting the 3D pose if the object lacks texture or color changes. Hinterstoisser et al. have proposed a method using gradient orientation to detect 3D textureless objects in real time under heavy background clutter, illumination changes and noise [4]. Although this state-of-art method could estimate the pose very fast and is robust to occlusion, it requires parts to have the discriminative color changes with the background. For textureless shiny objects, Rodrigues et al. have used a multi-light imaging system and random ferns to map the patch appearance into pose hypotheses votes to estimate the pose [5]. Shroff et al. have developed a multi-flash camera to extract specular features to find needles [6]. These specular features are captured in two or three positions and could identify line correspondence across views, which reconstruct a screw axis as a 3D line. Even though these methods [5,6] do not require the object to have texture, they can only be used for specular parts, and they also require changes to the lighting environment, which necessitates more complex system hardware. Liu et al. have proposed the Fast Directional Chamfer Matching method, which can perform pose estimation in 1 s [7]. However, this method is highly dependent on the depth edge, a geometric feature, estimated by a multi-flash camera, which is out of production. A hierarchical view-based approach using a single camera image has been proposed by Ulrich et al. [8]. This method takes the scale-space effect into account for the model image generation and searches the correct pose in a tree structure, which achieves a high recognition rate and a short computation time.

**Figure 1:**
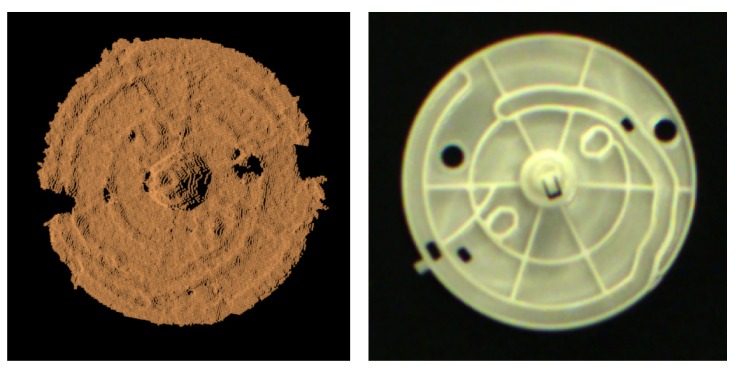
Defective point cloud of Part A (Left: The captured point cloud of the part; Right: The appearacne of the part). The detailed point cloud of the ridges in the part cannot be captured with the embedded 3D sensor algorithm. Thus, some previous methods fail to estimate the rotation around the center.

Moreover, 3D key point descriptors are used for pose estimation [9,10,11,12] with 3D inputs such as point clouds and depth images. Aldoma et al. [11] have proposed computing the clustered viewpoint feature histogram descriptor using a smooth region growing algorithm in order to conduct pose estimation in a short time. However, it relies heavily on segmentation and does not show good performance for industrial parts. To obtain a better performance for industrial parts, Drost et al. have proposed a state-of-the-art pose estimation algorithm using 3D point clouds [12]. They have combined a voting scheme and Point Pair Feature (PPF), which enables pose estimation of industrial parts in a clustered scene regardless of occlusion. Wu et al. have constructed a real bin picking system using the method proposed by Drost et al. for pose estimation [13]. However, the performance of this method [12] drops in the presence of many outliers and has bad performance in normal estimation, as stated in [14]. To solve the problem caused by outliers, Birdal et al. have coupled object detection with segmentation, where each segment is subject to disjoint pose estimation [15]. Applying a weighted Hough voting and an interpolated recovery of pose parameters, all the generated hypotheses are tested via an occlusion-aware ranking and sorted. With this combination, they have increased the detection rate and the accuracy of the resulting pose while reducing the complexity of the algorithm. However, this method is still highly dependent on the estimation of the normal. For the part shown in Figure 1, the normal calculated from the point cloud of a scene is different from the normal calculated from the model point cloud. This is because the captured points near the ridge differentiate the real appearance of the part. The ridges are 3 mm thick on this part, too thin to be captured by popularly-used commercialized 3D sensors. Because the 3D information of the plane surface on the part is sufficient, but near ridges is insufficient, the pose can be calculated except the horizontal rotation.

Some pose estimation algorithms use 2D (image) and 3D (point cloud) information together, as in the RDG-D image or organized point cloud. Jay et al. have presented a pipeline consisting of convolutional neural network-based segmentation for images and a 3D point cloud alignment pose estimation that can be processed in real time [16]. Hinterstoisser et al. have generated model templates with synthetic rendering of the object and performed pose verification using color and depth information [17]. This method is considered state-of-the-art, but nonetheless, it cannot be used for parts like Part A piled up in a bin because it does not have obvious color changes in the bin.

To solve the above-mentioned problems, we propose a Point Pair Feature (PPF)-based descriptor named Boundary-to-Boundary-using-Tangent-Line Boundary (B2B-TL) PPF, to allow accurate and quick bin picking of parts, although its point cloud is defective. The proposed descriptor utilizes the 3D point cloud data and 2D image data of the scene simultaneously, where the 2D image data could compensate the missing key details of the point cloud. In addition, we improve the manner of constructing the model description by using Multiple Edge Appearance Models (MEAM). Our method reduces the useless point pair information, thus decreasing online computation time and increasing the recognition rate. A pose estimation pipeline is proposed to leverage the usage of B2B-TL and MEAM. Please note that this method is designed for the scenes, wherein only one type of part exists, which are common scenes in real factories.

The remainder of this paper is organized as follows. Section 2 introduces the overview of the proposed algorithm. Section 3 presents the definition of B2B-TL. Section 4 explains the model description with MEAM. Section 5 details each process in the online computation. Section 6 presents the results of the validation experiment conducted using the proposed method.

## 2. Overview of the Proposed Algorithm

**Figure 2:**
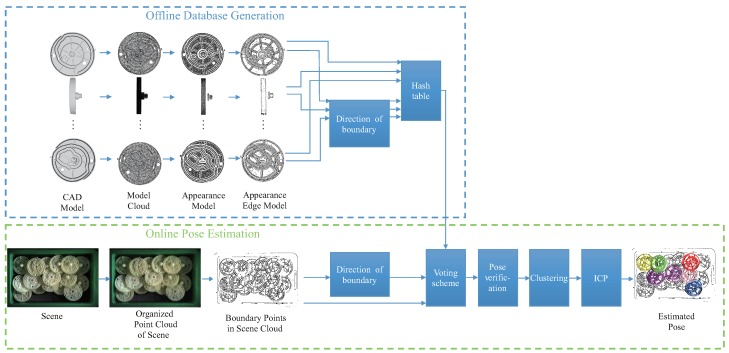
The full Point Pair Feature (PPF)-MEAM pipeline. This algorithm could be divided into two phases. In the offline phase, a database is constructed using the target model. In the online phase, the pose of target part is estimated using the organized point cloud of the scene.

We propose an algorithm capable of estimating the pose of industrial parts using an organized point cloud as the input. The pipeline of this algorithm is shown in Figure 2. Similar to the method proposed by Drost et al. [12], this technique can be divided into two phases: offline database generation and online pose estimation.

**Figure 3:**
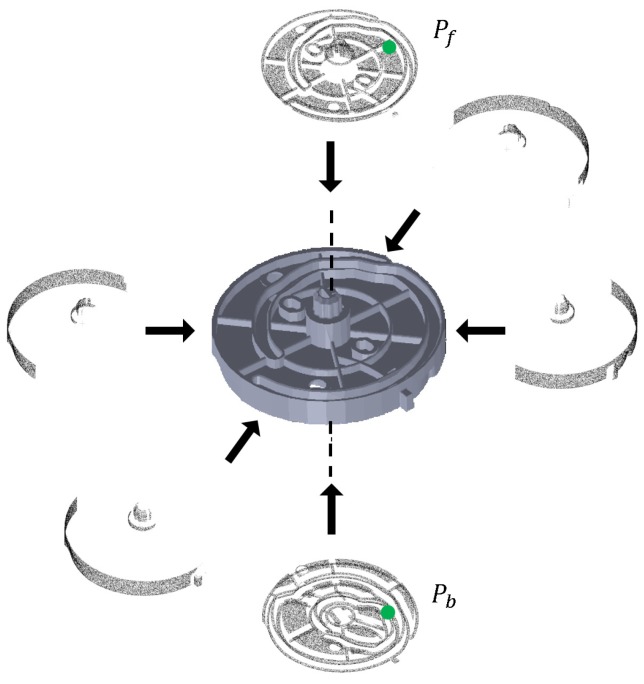
Visible points extracted from six view points. Points $P_{f}$ and $P_{b}$ are located at two different sides of the part, which cannot be seen simultaneously. However, this point pair ($P_{f}$, $P_{b}$) is calculated, and the PPF is stored in the hash table in other PPF-based methods.

The aim of offline database generation is to build a hash table that will be used as the database in the online phase. The point cloud is generated using the CAD model of the target part. Then, the visible points from *N* viewpoints are extracted from the model using the method proposed by Katz et al. [18]. Evidently, all points should be visible because the volume of a point is infinitesimal. However, once a surface is reconstructed from the points, it is possible to determine which of the points are visible. The result of the visible points extracted from *N* (N=6) viewpoints is shown in Figure 3. We name the *N* extracted models as *N* multiple appearance models. Next, for each appearance model, we perform the boundary points’ extraction using the method provided by the Point Cloud Library (PCL) [19]. We name the resulting model as the edge appearance model. Then, the B2B-TL of all point pairs in each edge appearance model is calculated and stored in one hash table. Please note that this paper does not consider a boundary to differ from an edge.

Given the organized point cloud of a scene, the pose of the part is estimated in the online phase. The organized point cloud is captured by a color camera sensor and a 3D sensor. The relative transformation between the camera and 3D senor is calibrated in advance. Because the boundary extraction method used for the model depends highly on the normal of each point, it cannot be used for parts such as Part A. Thus, we perform the Canny edge algorithm [20] on the grayscale images, images converted from color images captured by a color camera, to enable edge detection and locate the boundary points by mapping the 2D edge pixels and 3D point cloud.As the relationship between the color camera sensor and 3D sensor is calibrated, this process is easy. Then, the B2B-TL is calculated for the edge points of the scene, and a voting scheme similar to that mentioned by Drost et al. [12] is performed to obtain multiple coarse pose candidates. Next, the pose verification proposed by Li et al. [21] is performed to remove the wrong pose candidates. Unlike Li’s method, we perform the pose verification using MEAM. For each model, we could obtain a defined number of pose candidates. We then cluster these pose candidates and choose the pose with the highest pose verification score to represent each cluster. Next, the Iterative Closest Point (ICP) algorithm [22] is performed to refine the pose candidates. Finally, the pose result is output and sent to the grasp planning process to generate the final grasp pose.

## 3. Boundary-to-Boundary-using-Tangent-Line

The B2B-TL is a novel descriptor based on the 3D points on the boundary of an object, which are used to find the correspondences between the scene and the model by matching their descriptors. Points on the boundary hold a considerable amount of essential information about the shapes of the objects. Therefore, only a few points are needed to differentiate objects, leading to highly precise pose estimation. After we obtain the boundary points, we use the direction of the tangent line, instead of the normal, as the orientation. This leverages the points whose normal could not be estimated accurately, but can be used for estimating the pose for the parts, like Part A and Part C, as shown in Figure 8. In addition, this feature generalizes feature $F_{B2B}$, which has been introduced by Choi et al. [23]. The $F_{B2B}$ is designed for planar objects, which have straight line boundaries. We improve upon it to enable it to work with more complex parts, such as Part A, Part B and Part C, which mainly consist of arc boundaries.

To obtain the boundary point of the parts, we use a vision sensor set consisting of a 3D sensor and a color camera. We first convert the color image to the grayscale image to perform the Canny edge algorithm for edge detection. Then, we map these edge pixels to the points in the point cloud. The corresponding points are extracted as the boundary point cloud.

The boundary points are then divided into many ROIs in preparation of calculating the tangent line for each point. Algorithm 1 shows how to divide the ROIs, and Algorithm 2 shows how to calculate the tangent line. We use the coordinate system as shown in Figure 10a to calculate the projection of 3D points.Both directions of the tangent line would be used as the orientation of each point.

**Algorithm 1:** Divide the points of a scene into many ROIs.

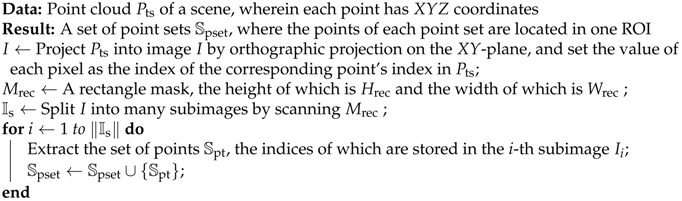



**Figure 4:**
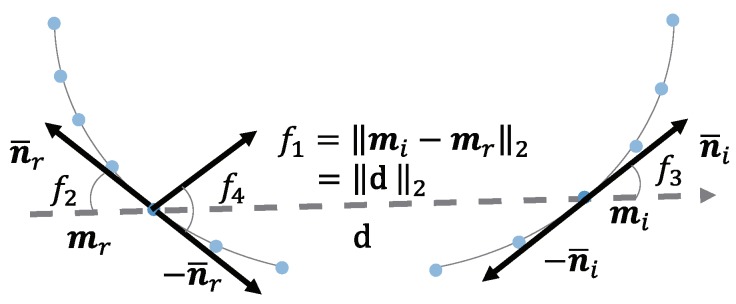
The definition of the B2B-TL feature. This feature is different from the other PPF because it is using the points not only on the line segments, but also on the curves. A line cannot be fitted for the point on a curve, but the tangent line can be calculated and its direction used as the direction of the point.

After calculating the direction for each point, we calculate the $F_{B2B-TL}\in R^{4}$ feature. The feature is shown in Figure 4. $m_{r}$ and $m_{i}$ are the reference and referred points on the boundary of an object, and ${\bar{n}}_{r}$ and $-{\bar{n}}_{r}$ are the directions of point $m_{r}$, while $-{\bar{n}}_{i}$ and ${\bar{n}}_{i}$ are the directions of $m_{i}$. The feature is given by:

(1) $$\begin{array}{cc} F_{B2B-TL} & ={\left(f_{1},f_{2},f_{3},f_{4}\right)}^{⊤}\\ & ={\left({∥d∥}_{2},∠\left({\bar{n}}_{r},d\right),∠\left({\bar{n}}_{i},d\right),∠({\bar{n}}_{i},{\bar{n}}_{r}\right)}^{⊤}, \end{array}$$

where **d** is the vector from the reference point to the referred point and $∠\left(v_{1},v_{2}\right)\in \left[0,\frac{\pi }{2}\right]$ represents the angle between two vectors. The first component of this feature, $f_{1}={∥m_{r}-m_{i}∥}_{2}={∥d∥}_{2}$, represents the Euclidean distance between the two points. The second and third components, $f_{2}$ and $f_{3}$, are sharp angles between the vector **d** and tangent lines that pass through point $m_{r}$ and point $m_{i}$, respectively. The fourth component $f_{4}$ is the sharp angle between these two tangent lines. Considering that the direction of tangent lines that pass each point could be ${\bar{n}}_{r}$ or $-{\bar{n}}_{r}$ simultaneously, we only utilize the sharp angle of $∠\left({\bar{n}}_{r},d\right),∠\left({\bar{n}}_{i},d\right),∠\left({\bar{n}}_{i},{\bar{n}}_{r}\right)$. Similar to the method proposed by Drost et al. [12], the distances and the angles are sampled in steps of $d_{\mathrm{dist}}$ and $d_{\mathrm{angle}}$.

**Algorithm 2:** Calculate the tangent line direction for each point.

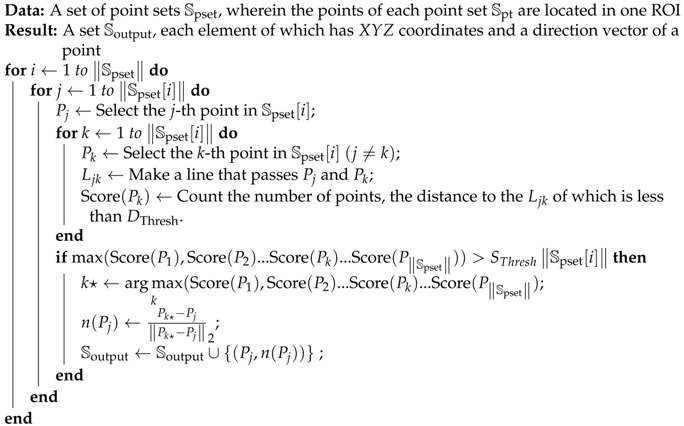



## 4. Offline Computation Process: Model Description with MEAM

We describe the target object using a set of selected pair features computed from MEAM. Once the pair features in the scene point cloud are calculated, the pose of the object can be estimated by matching the pair features of the scene with the set of the model pair features. We construct the representation of a target object using the proposed descriptor offline. The representation of the model is implemented in a hash table. The pair features are quantized and used as the key for the table so that similar point pairs are inserted in the same bin.

Unlike previous research, as seen in [12,23], our algorithm uses MEAM instead of the original model (including the points of all sides). There are reasons to use MEAM rather than one model consisting of all points for the part. In the method proposed by Drost et al. [12], every two points are treated as a point pair, for which a PPF is calculated and saved in the hash table. However, some points would never be seen in a scene, as shown in Figure 3. With such unrealistic point pairs (points on two inverse sides) in the hash table, some wrong votes may be cast to some wrong pose candidates during the voting scheme, which results in wrong pose estimation. In addition, such point pairs increase the hash table size and online computation time, because it must be considered when they have the same PPF as the point pairs in the scene. The same problem arises with the method proposed by Choi et al. [23]. However, using multiple appearance models could solve the problem. Furthermore, as shown in Figure 3, the normal directions of $P_{f}$ and $P_{b}$ are inverse because they are located on the inverse sides of the part. In fact, the existence of two points’ normal direction in the inverse direction in a scene is rare. However, when the fitted tangent line is used as the point’s direction, having points with the direction being a nearly inverse direction is not rare. This necessitates making point pairs using only points of appearance models.

**Figure 5:**
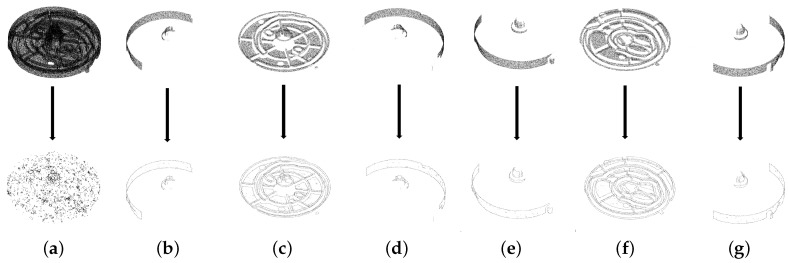
Comparison between the boundary extraction using the original model and multiple appearance models. (**a**) shows extracted boundary points using the original model, that is, the model with all points around the part. (**b**,**c**,**d**,**e**,**f**,**g**) show extracted boundary points using multiple appearance models. Multiple appearance models outperform the original model in terms of boundary extraction.

Moreover, using visible points makes boundary estimation easier for the model. Because the model point cloud is generated from the CAD model, it does not have noisy points and does not suffer from the deficiency caused by changes in light as in the point cloud of scene. Therefore, we prefer estimating the boundary using only the model point cloud, but not rendering the part and performing edge detection as we do for the scene. However, the boundary estimation only works well while using multiple appearance models rather than a model with points of all sides. The boundary estimation is based on the method proposed by Radu et al. [24] and is a well-used tool in the PCL [19], which recognizes the boundary points by the gradients of *x*, *y* and *z* and the direction of the normal, of a point. The boundary estimation fails for Part A while using the original model because the accurate normal of a point is difficult to estimate, especially for points on the thin ridge of Part A. Moreover, determining the angle criterion for the gradient of the normal raises another question. We posit that the thin ridges on the surface of the part raise difficulties when using existing methods. A multiple appearance model provides better performance because not every appearance model has points on the planes perpendicular to visible planes. This makes it easier to locate boundary points by checking the gradients of *x*, *y* and *z* of a point, as shown in Figure 5.

Given a model point cloud, *N* viewpoints are selected around the object, and consequently, *N* appearance models are extracted. We chose six (N=6) viewpoints, that is a small subset of endless arbitrary viewpoints in a real situation. However, all points in the model are considered. Many viewpoints are used in the method proposed by Drost et al. [25], but some visible point models do not differ much, which reduces the accuracy and increases the computation time. The visible points’ extraction is based on the method proposed by Katz et al. [18]. For each appearance model, the PPFs of every two points are calculated and saved into one hash table. In order to record the point pair and to which model it belongs, each point in the six models has a unique index. The point pairs with the same feature are saved in the same slot, and the B2B-TL is used as the key, which is similar to Drost’s method.

**Figure 6:**
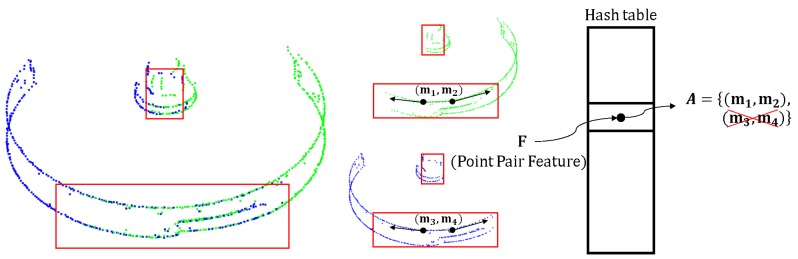
Save the same point pairs only once in the hash table. The appearance edge models in green and blue are extracted from different viewpoints. These two appearance edge models share some of the same points, as shown in the red box. The point pair $\left(m_{1},m_{2}\right)$ and $\left(m_{3},m_{4}\right)$ is the same point pair in reality, but their points have different indices because they belong to different appearance models. Using the proposed encoding method, we recognized that these two point pairs are located at the same position and that only one pair needed to be saved in the hash table.

However, this method is problematic when the same point pairs are saved into the hash table, because different appearance models share some same point pairs. As shown in Figure 6, there are two example appearance edge models in green and blue that were extracted from different viewpoints. These two models share some same points, which are shown inside the red boxes, because you can see some of the same regions of the part even if you change your viewpoint. The points in such regions are saved in these two appearance edge models repeatedly. When we calculate the B2B-TL for points in each appearance model and save it in the hash table, the points in the sharing regions would be used twice. This increases the computation time, as some repeated and redundant point pairs need to be calculated in the voting scheme.

Therefore, as another unique proposition, we remove the repeated point pairs by encoding each point pair of *N* models. To identify the repeated point pairs, we could not use the index of the points in the point pair. For example, $m_{1}$ and $m_{3}$ in Figure 6 have different indices because they belong to different edge appearance models, though their *X*, *Y* and *Z* coordinates are the same. To identify the point pair, we encode two points in the pair first and combine their codings. The point can be encoded using its *X*, *Y* and *Z* coordinates. However, the values of coordinates are floating, and we alternatively use the index of the voxel, the three-dimensional analogue of a pixel, to which the point belongs. The voxel space, a three-dimensional space with grids, is established for the original model (the model with the points of all sides), as proposed by Li and Hashimoto [21]. Thus, each point is represented by a 3D vector. Elements of this vector are the index of the voxel with respect to the *x*, *y* and *z* axes. Given a point $p_{i}$, the *X*, *Y*, and *Z* coordinates values of which are $x_{i}$, $y_{i}$ and $z_{i}$, respectively, its representation in the voxel space can be calculated by Equation (2):

(2) $$\begin{array}{c} x_{ind}=\mathrm{INT}\left(\frac{x_{i}-X_{\min}}{L_{\mathrm{voxel}}}\right)\\ y_{ind}=\mathrm{INT}\left(\frac{y_{i}-Y_{\min}}{L_{\mathrm{voxel}}}\right)\\ z_{ind}=\mathrm{INT}\left(\frac{z_{i}-Z_{\min}}{L_{\mathrm{voxel}}}\right), \end{array}$$

where $X_{\min},Y_{\min}$ and $Z_{\min}$ are the minimum coordinate components of the model cloud; $x_{ind},y_{ind}$ and $z_{ind}$ are the indices of each axis; and $L_{\mathrm{voxel}}$ is the length of the voxel. For example, we use vector $m_{1}=\left(1,2,3\right)$ and vector $m_{2}=\left(4,5,6\right)$ to represent these two points in the voxel space, then the point pair $\left(m_{1},m_{2}\right)$ is encoded by c=(1,2,3,4,5,6). Because the $\left(m_{3},m_{4}\right)$ is located in the same position, it is also encoded by c=(1,2,3,4,5,6). With this encoding method, we remove the repeated point pairs from the hash table as shown in Figure 6.

## 5. Online Computation Process

In the online phase, the sensor captures the organized point cloud of a scene with a camera and a 3D sensor. By performing the edge detection, the edge pixels are detected in the 2D image. Mapping the edge pixels to 3D points, we obtain the boundary points in the scene cloud. Using the algorithms shown in Algorithms 1 and 2, we calculate the direction of each point. Next, we perform the voting scheme, a commonly-used approach in the PPF-based algorithm, using the trained database to obtain coarse pose candidates. The following pose verification removes the wrong pose candidates. The remaining pose candidates are sorted by the score of the pose verification, and some similar pose candidates are removed by clustering. The poses are further refined by the ICP algorithm. The following sections provide the details of each step.

### 5.1. Point Pair Feature Voting Scheme

**Figure 7:**
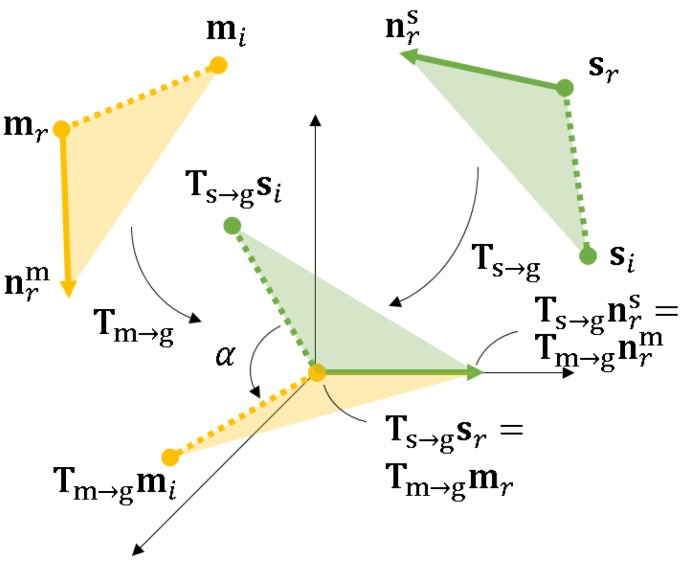
Transformation between the model and scene coordinates. $T_{s\to g}^{-1}$ transforms the scene reference point $s_{r}$ to the origin and aligns its direction $n_{r}^{s}$ to the *x*-axis of the intermediate coordinate system. The model reference point $m_{r}$ and its direction $n_{r}^{m}$ are transformed similarly by $T_{m\to g}^{-1}$. Rotating the transformed referred scene point $T_{s\to g}^{-1}s_{i}$ with angle α around the *x*-axis aligns it with the transformed referred model point $T_{m\to g}^{-1}m_{i}$. ($m_{r}$, α) is then used to cast a vote in the 2D space.

In this paper, we use a voting scheme conducted in the 2D space using local coordinates, as proposed by Drost et al. [12]. Given a scene point pair $\left(s_{r},s_{i}\right)$, its B2B-TL is calculated and used as the key to find the corresponding point pair $\left(m_{r},m_{i}\right)$ in the hash table. An improvement is proposed in that if the distance between the two points $\left(s_{r},s_{i}\right)$ is longer than $d_{\max}$, the longest distance in the model, then this point pair will not be considered in later steps. We then align the reference points of pairs $s_{r}$ and $m_{r}$ at the origin of an intermediate coordinated system, as shown in Figure 7. Their tangent lines $n_{s}^{r}$ and $n_{m}^{r}$ are aligned to the *x*-axis. The referred points $s_{i}$ and $m_{i}$ are aligned by rotating around the *x*-axis. The rotation angle α and the reference model point compose the local coordinate $\left(m_{r},\alpha \right)$. The transformation from $\left(m_{r},m_{i}\right)$ to $\left(s_{r},s_{i}\right)$ is calculated with the following equation.

(3) $$\begin{array}{cc} s_{i} & =T_{s\to g}^{-1}R_{x}\left(\alpha \right)T_{m\to g}m_{i}, \end{array}$$

where $T_{s\to g}^{-1}$ and $T_{m\to g}$ are the transformation from the scene and model coordinate systems to the intermediate coordinate system, respectively. $R_{x}\left(\alpha \right)$ is the rotation around the *x*-axis with angle α.

Given a reference scene point $s_{r}$, a defined percentage of every other point $s_{i}$ is paired. The model point pairs $\left(m_{r},m_{i}\right)$, which have the same B2B-TL feature as the scene pair feature $\left(s_{r},s_{i}\right)$, are extracted from the hash table. For every correspondence between $\left(m_{r},m_{i}\right)$ and $\left(s_{r},s_{i}\right)$, the angle α is calculated. The votes are finally cast in the 2D space of $\left(m_{r},\alpha \right)$. For the local coordinate with the highest vote, the transformation between the model and scene coordinate systems is computed using Equation (3). The same process is repeated for every other reference scene point. As a result, each reference scene point corresponds to one pose candidate, which is recorded. We also record the vote score, the reference appearance edge model for a later stage in the process.

### 5.2. Pose Verification

Pose verification is then conducted to remove the wrong pose candidates after the voting scheme. The pose is verified by transforming the model cloud into the scene space. For every transformed model point, the nearest scene point is searched, and the distance between these two points is computed. The transformed model point is considered as the fitted point if the distance that is calculated in the voxel space is smaller than a given threshold. The pose is considered to be correct if the number of the fitted points is sufficiently large. The details of this method are discussed by Li and Hashimoto [21].

When we apply MEAM, the pose verification process differs slightly. After the voting scheme is complete, every pose candidate corresponds to one model reference point. Therefore, we could track the model from which the point originates. Instead of using the original model point cloud, which includes all points from every side, the corresponding appearance edge model is transformed to the scene. This improves the performance of pose verification for parts such as Part A, Part B and Part C, in which the top side of the object is similar to its bottom side, as shown in Figure 3. For example, when using the original model point cloud, cases pertaining to the wrong pose (which makes the bottom side of the transformed model point cloud align with the top side of the object) may attain a high score. On the other hand, if we use an appearance model with only the points of the top side of the object, the score of the pose will be very low. Consequently, this helps remove the wrong pose candidates.

All poses are sorted based on their pose verification score. For each appearance model, we choose the top $N_{\mathrm{pv}}$ pose candidates. These pose candidates are then clustered. For each cluster, we only retain the pose with the highest pose verification score and remove the rest. The output of these pose candidates is finally sorted by their pose verification scores.

The part without any occlusion tends to have a high pose verification score, because it has more points than parts with occlusion. Because of this advantage, the rank of the part corresponds to the extent of this occlusion. This information is very important when we perform the picking task and achieve a defined placement, because it is better to grasp the part without occlusion. Therefore, we retain this particular order of pose candidates for the grasp planning process.

### 5.3. Pose Refinement

After pose verification, we refine the remaining pose candidates using the ICP algorithm [22]. Similar to the process in the pose verification step, we use the corresponding appearance model to perform the ICP algorithm for each pose candidate. This offers an advantage similar to that stated in the pose verification section. Using the original model point cloud may align the bottom of the object with its side in the scene. Hence, using MEAM leverages the ICP algorithm to refine the pose candidates.

## 6. Evaluation Experiment

**Figure 8:**
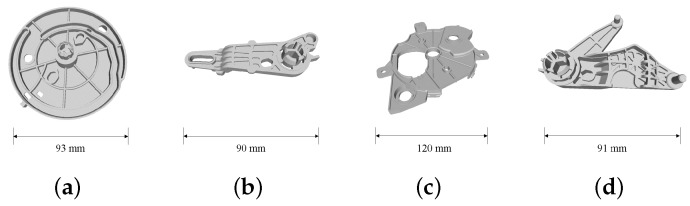
Industrial parts used to verify the proposed method. We named these parts as Part A (**a**), Part B (**b**), Part C (**c**) and Part D (**d**). They are made of resin and used in a real car air-conditioning system. The appearance of these parts is complex, making the pose estimation more difficult compared to cases in which the parts have primitive shapes.

In this section, we evaluate the proposed method using synthetic and real scene data with the parts, as shown in Figure 8. The picking task was also conducted to estimate the performance of this method in a practical application.

For all experiments, we subsampled the boundary points of the scene cloud with a leaf size of 1 mm. $H_{\mathrm{rec}}$ and $W_{\mathrm{rec}}$ were set to be 10 pixels. We set $D_{\mathrm{thresh}}$ and $S_{\mathrm{thresh}}$ to 0.3 mm and 0.18. The $L_{\mathrm{voxel}}$ that was used to build the voxel space was set to 1. The $\tau_{d}$ that makes $d_{\mathrm{dist}}=\tau_{d}\times d_{\max}$ was set to 0.07. The $d_{\mathrm{angle}}$ was set to $3.6^{\circ }$ according to our experience.

The algorithm was implemented in C++ and run on an Intel Core i7 6950X CPU (Intel Corporation, Santa Clara, CA, USA). Because the parallelization of pose estimation program is frequently implemented in practical application in factories, the parallelization of the proposed algorithm and the PPF method proposed by Drost et al. [12] was realized based on OpenMP to evaluate its extreme performance in the real application. To make sure that the parallelization was conducted on ideally the same level, we used the program of the contrast algorithm implemented by us, but not the one implemented by the original author. Note that the proposed method uses the 3D point cloud and image as the input and the contrast algorithm uses the 3D point cloud only.

### 6.1. Synthetic Data

**Figure 9:**
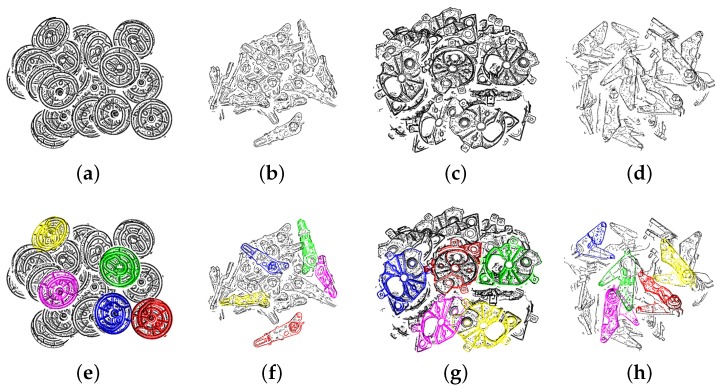
(**a**,**b**,**c**,**d**) are the simulated scenes of the example parts. We estimated 5 poses in each scene, and (**e**,**f**,**g**,**h**) are the results of the pose estimation. The model point cloud is transformed to the scene space using the pose estimation results and rendered with different colors. These colors indicate the recommendation rank to grasp the part after considering occlusion. Models 1–5 are rendered in red, green, blue, yellow and pink, respectively.

To estimate the performance of the proposed method, we generated 30 scenes for 4 types of target parts. For each scene, we simulated 20 target parts that drop down one by one from a defined position above a bin with a random orientation. We assumed that the 2D boundary extraction was perfect, so as to be able to use the edge appearance model to create the synthetic data. To approximate the real scene, we assigned a viewpoint to view these parts and only used the visible points from that viewpoint. Four examples of synthetic scenes are shown in Figure 9.

The estimated pose was compared with the ground truth. If the errors in translation were within $10\%\times d_{\max}$ and in rotation within $5^{\circ }$, we counted the case as a true positive; otherwise, it was regarded as a false positive. From our experience of using the bin-picking system, the error of pose within this range is acceptable for the robot to grasp. The recognition rate equals the fraction of the number of true positives among the number of output poses.

For different parts, some parameters need to be tuned to obtain a good balance between the performance of recognition rate and computation time. $P_{\mathrm{ref}}$ and $P_{\mathrm{referred}}$ are two parameters for this trade-off. $P_{\mathrm{ref}}$ is the percentage of the scene boundary points used as the reference points, while $P_{\mathrm{referred}}$ is the percentage of the scene boundary points used as the referred points. $P_{\mathrm{referred}}$ is set to 20%. $P_{\mathrm{ref}}$ is set to 5%, 20%, 5% and 10% for Part A, Part B, Part C and Part D.

The recognition rate and speed of the algorithms for every model are presented in Table 1 and Table 2, respectively. We compared our method with our own implementation of Drost et al. [12], namely original PPF. For parts such as Part A, the proposed method provided an estimation with a high recognition rate, while the original PPF did not work as precisely. In both algorithms, we used OpenMP to speed up the computation. The proposed method outperforms the original PPF [12] in terms of the recognition rate and computation time, as shown in the table below.

### 6.2. Real Scene Data

#### 6.2.1. Quantitative Evaluation

**Figure 10:**
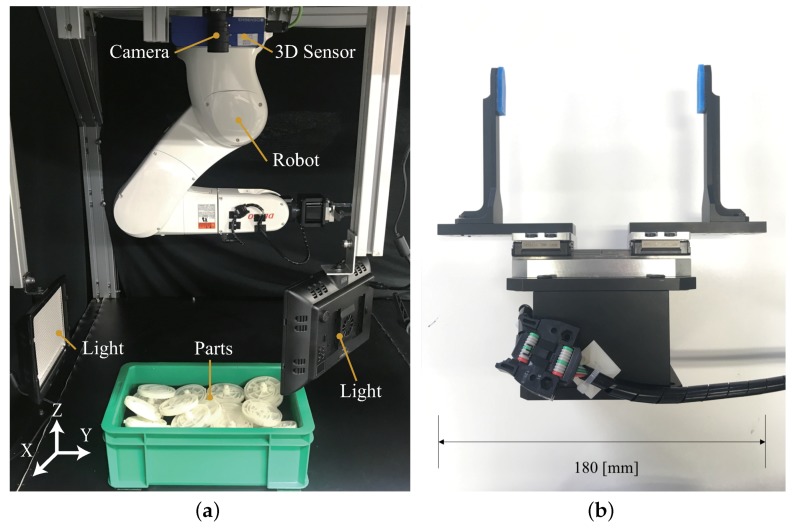
The experimental system (**a**) was used to verify the proposed method. A color camera and 3D sensor were mounted on top (above the parts). To mitigate the effect of shadow on edge extraction, we installed two Light-Emitting Diodes (LEDs) on both sides of the box. A robot was used to perform the picking task with the gripper, as shown in (**b**).

**Figure 11:**
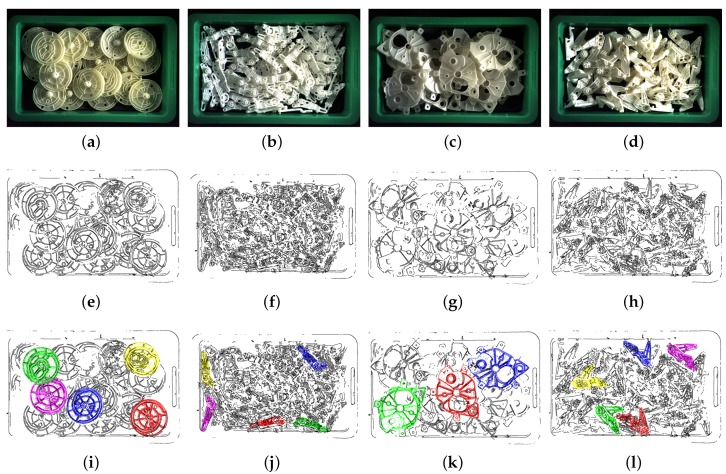
(**a**,**b**,**c**,**d**) are the real scenes of the example parts. (**e**,**f**,**g**,**h**) are the boundary points of the scene cloud. (**i**,**j**,**k**,**l**) are the results of pose estimation. The model point clouds are transformed to the scene space using pose results and rendered with different colors. These colors indicate the recommendation rank to grasp the part after considering the occlusion. Models 1–5 are rendered as red, green, blue, yellow and pink, respectively.

We tested 20 scenes for each type of part using original PPF [12] and our method. The real data were captured by a color camera and 3D sensor (Ensenso N35, Ensenso GmbH, Freiburg, Germany), and the whole system is shown in Figure 10a. The ground truth poses of the parts were identified manually. We detected 5 parts for Part A, Part B and Part D in each scene and 3 parts for Part C in each scene. That is because the size of Part C is bigger and only a few parts can be observed in each scene without occlusion. The true positives of each scene were counted to calculate the recognition rate. Some examples are shown in Figure 11. We rendered the top pose results in the same manner as the simulation result. $P_{\mathrm{referred}}$ is set to 20%. $P_{\mathrm{ref}}$ is set to 10%, 10%, 10% and 20% for Part A, Part B, Part C and Part D.

The recognition rate and algorithm speed for every model are presented in Table 3 and Table 4, respectively. The proposed method outperforms the original PPF [12] in terms of the recognition rate and computation. Because the correct normal of the part like Part A is too difficult to estimate in the real scene, as we pointed out in Figure 1, the performance of original PPF was not high. However, the proposed method used the B2B-TL and MEAM, which obtained a high success rate. Moreover, by comparing the success rate of PPF between Table 1 and Table 3, it is clear that the noise of the real scene point affected its performance. Nonetheless, the proposed method showed its robustness to the noise. Furthermore, thanks to the robustness of the proposed method, using points only on boundaries was enough to estimate the pose of the part, so that it took less time than original PPF.

#### 6.2.2. Qualitative Evaluation

**Figure 12:**
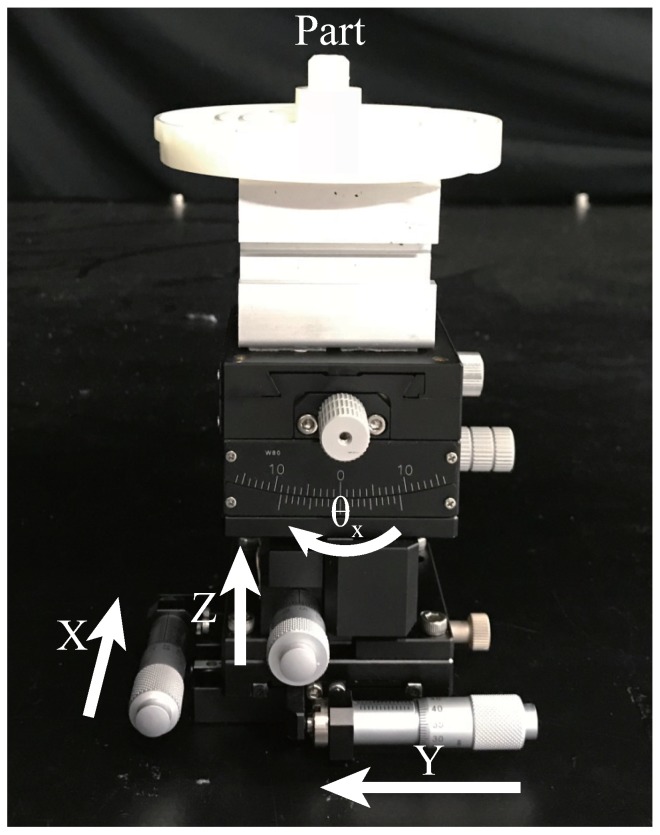
The multi-axis stage unit is used to evaluate the relative error of pose estimation. After fixing the part on the top of the stage, we could move the part along each axis with a precision of 0.01 mm and rotate it by $\theta_{x}$ with a precision of $0.1^{\circ }$.

**Figure 13:**
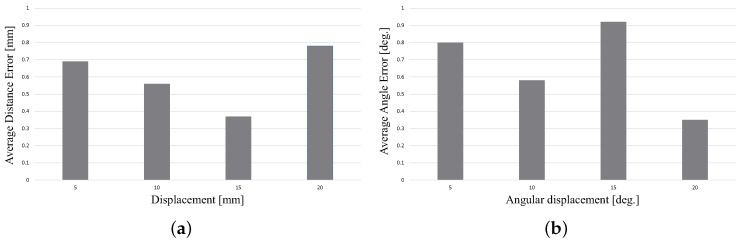
(**a**,**b**) are the results of the relative error experiment. The part is moved by 5 mm, 10 mm, 15 mm and 20 mm using the stage. Pose estimation was performed each time we moved it. The moved distance was calculated by comparing the differences in the pose results. We conducted 10 trials for one part, and the average distance error is shown in (**a**). Similarly, we rotated the part by $5^{\circ }$, $10^{\circ }$, $15^{\circ }$ and $20^{\circ }$, and the corresponding results are shown in (**b**).

To evaluate the pose error for each part, we used a multi-axis stage unit (Misumi Group Inc., Tokyo, Japan) as shown in the Figure 12, to conduct this experiment.When we fixed an object on this stage, we could move the object by a determined distance with a precision of 0.01 mm or rotate the object by a determined angle with a precision of $0.1^{\circ }$. We estimated the pose for Part A at an initial position. We then moved the part by a specified distance, namely 5 mm, 10 mm, 15 mm and 20 mm, and estimated its respective pose. The error between the moved distance and calculated distance was then computed (the distance can be calculated by comparing the current and initial poses). We repeated this process 10 times, and the average error of translation was obtained. The error of orientation was obtained similarly. After we estimated the part at an initial position, we rotated it by a specified angle, namely $5^{\circ }$, $10^{\circ }$, $15^{\circ }$ and $20^{\circ }$, and estimated its respective pose. Because of the limitation of the structure of this stage, we estimated the error of translation and orientation separately. The results are shown in Figure 13. Because it is very difficult to obtain the absolute pose, we used an indirect way to show that the pose error attained with our method is small enough for a bin picking system.

#### 6.2.3. Selection of the Number of MEAM

To confirm that using six MEAM could achieve a high performance, we conducted a comparison experiment for different *N*, the number of MEAM. We performed the pose estimation for 20 scenes of Part A using different *N*. The result is shown in Table 5.

When *N* was smaller than six, it took less computation time, but it also reduced the recognition rate. The reason is that MEAM does not cover all the faces of the part. When *N* was bigger than six, it took greater computation time because we needed to consider more corresponding MEAM in the pose verification step and pose refinement step. Its recognition rate dropped marginally because unrealistic point pairs (points on two inverse sides) were stored in the hash table; that is, because the visible points’ extraction method [18] in the offline phase has flaws in reliably extracting visible points. A few of the points on the invisible sides were extract, as well. With a large number of MEAM, the total number of unrealistic point pairs was increasing in the hash table, which consequently reduced the recognition rate. For these reasons, we show that six MEAM could achieve a high performance of pose estimation.

#### 6.2.4. Advantage of using MEAM

To show the advantage of using MEAM, we conducted a comparison experiment, wherein we performed the pose estimation for 20 scenes of Part A using 3 methods. The result is shown in Table 6. The first method was the proposed method. The second method was based on the B2B-TL, but we used the boundary points of the original model to build the hash table. In this method, the step shown in Figure 6 was not conducted. Thus, it took longer and resulted in a lower recognition rate than the proposed method. The third method was the original PPF [12]. The comparison shows that our proposed method can achieve higher recognition rates and result in less computation time.

### 6.3. Verification on Tohoku University 6D Pose Estimation Dataset

To allow other researchers to be able to compare their methods with ours, the Tohoku University 6D Pose Estimation Dataset [26] was created. This dataset differs from other datasets [27,28] because it focuses on the scenes wherein only one type of industrial part is randomly piled up in a bin. To the best of our knowledge, such scenes are much more common than the scenes wherein multiple types of parts are mixed together because of aspects like batch production, storing and the Kanban system.

**Figure 14:**
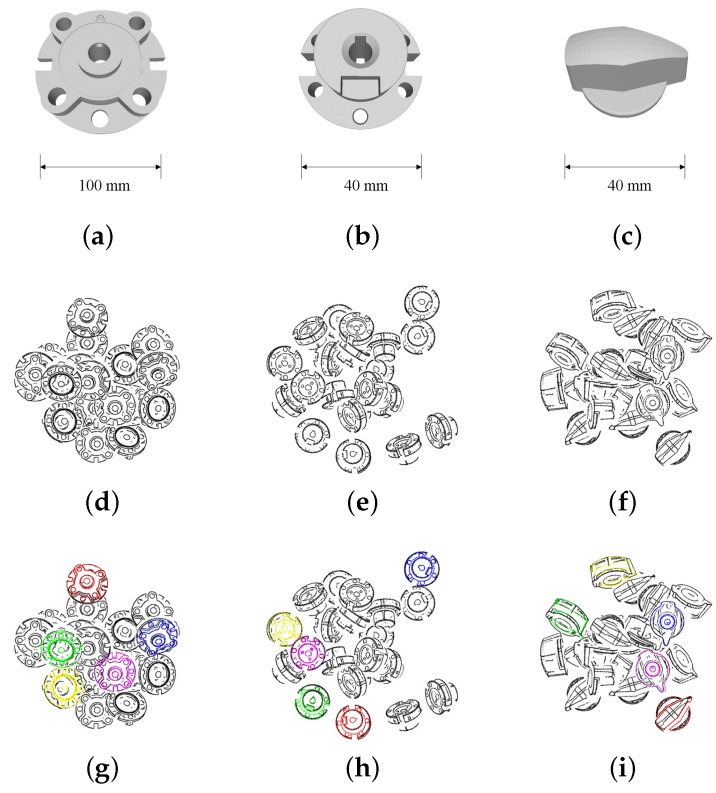
Industrial parts in the Tohoku University 6D Pose Estimation Dataset. (**a**,**b**,**c**) are named as Part 1, Part 2 and Part 3, respectively. The example synthetic scenes are shown in (**d**,**e**,**f**). (**g**,**h**,**i**) are the results of the pose estimation.

The currently available synthetic scene data of three types of parts in this dataset were used to test our method and the one proposed by Drost et al [12]. The tested parts and the synthetic scenes are shown in the Figure 14. The result is shown in Table 7 and Table 8.

In this experiment, we used the same method to calculate the recognition rate as mentioned in Section 6.1. In this experiment, our method outperformed original PPF in terms of the average recognition rate and average computation time. However, the difference was not as huge as the one shown in Table 1 and Table 2. The reason is that the normal of these parts, which have few ridges on their surface, was easier to estimate than the parts shown in Figure 8. For Part 3, the performance of Original PPF was higher than ours. This shows that our method had a limited improvement when there were some curvy surfaces or few robust edges on the object.

Because parts shown in Figure 8 are being used in real products, we were not allowed to publish the details of these parts. Alternatively, similar parts are being prepared, and their synthetic scene and real scene data are going to be published in this dataset soon. Researchers are welcome to upload their experimental results to show the comparison with our method.

### 6.4. Bin-Picking System Performance

To estimate the performance of our method in a real situation, we integrated it with a 6-DoF manipulator system. The name of the manipulator is VS-068 (DENSO WAVE INC., Kariya, Japan). We used the Ensenso N35 (Ensenso GmbH, Freiburg, Germany) as the 3D sensor and the iDS USB 3 uEye (IDS Imaging Development Systems GmbH, Obersulm, Germany) as the 2D camera. The name of the gripper is ESG 2 Series (TAIYO, LTD., Osaka, Japan). To make the bottom of the box was bright enough, and to avoid being affected by the surrounding light, 2 LEDs were set near the bin.

Multiple pieces of Part A were piled up randomly in the bin, as shown in Figure 10a. $P_{\mathrm{ref}}$ and $P_{\mathrm{referred}}$ were set to 10% and 20%. Six edge appearance models were used to build the hash table. All pose candidates after the voting scheme were verified. The top 5 pose candidates for each model were selected to conduct the cluster process. The pose candidates after the cluster step were refined using the ICP algorithm. We placed a total of 12 parts in the bin and picked them up until no parts remained inside it. If we failed in picking once, we estimated and picked it again. We tested 5 bins of parts as shown in Table 9, and our success rate was 95.2% over 63 trials, while the average computation time was about 889 ms for pose estimation. We failed in picking the part 3 times. Two times, the failure was because the gripper touched other parts before it closed, and then, it could no grip the part. We failed once because there was no result of grasp planning. We have attached a video showing the picking task using this system for one bin of parts.

## 7. Conclusions

In this paper, we propose a novel point pair feature-based descriptor named Boundary-using-Tangent-Line (B2B-TL) and a model description using Multiple Edge Appearance Models (MEAM). A pose estimation pipeline is then described using the B2B-TL and MEAM, which estimates the poses for industrial parts so as to retrieve the 6D pose. Our algorithm is evaluated against a large number of synthetic and real scenes, and the results indicated a high success rate of pose estimation, as well as lower computation time. A pose estimation dataset for industrial parts has been proposed for other researchers to compare their methods with ours. Finally, our method is integrated with a bin picking system, helping it attain a high picking success rate. The experimental results show that our method is sufficiently accurate for a robot to grasp industrial parts and is fast enough to be used in a real factory environment.

## Figures and Tables

**Table 1:** Recognition rate of the algorithms for synthetic scenes.

Methods	Part A	Part B	Part C	Part D	Average
Proposed Method	**100%**	**99.3%**	**100%**	**100%**	**99.8%**
Original PPF [12]	49%	66%	71.3%	90%	69.2%

**Table 2:** Speed of the algorithms for synthetic scenes (ms/scene).

Methods	Part A	Part B	Part C	Part D	Average
Proposed Method	**1276**	**401**	**987**	**892**	**889**
Original PPF [12]	3033	1470	1754	4430	2671

**Table 3:** Recognition rate of the algorithms for real scenes.

Methods	Part A	Part B	Part C	Part D	Average
Proposed Method	**97%**	**97%**	**98.3%**	**97%**	**97.2%**
Original PPF [12]	8%	72%	58.3%	89%	57%

**Table 4:** Speed of the algorithms for real scenes (ms/scene).

Methods	Part A	Part B	Part C	Part D	Average
Proposed Method	**1322**	**1353**	**1271**	**1360**	**1326**
Original PPF [12]	2752	3673	1765	1438	2407

**Table 5:** Comparison experiment for different *N*.

N	1	2	6	14
Recognition rate	69%	93%	**97%**	95%
Time (ms/scene)	**942**	1140	1322	3560

**Table 6:** Comparison experiment using Part A.

	Proposed Method	Proposed Method without MEAM	Original PPF [12]
Recognition rate	**97%**	91%	8%
Time (ms/scene)	**1322**	2710	2752

**Table 7:** Recognition rate of the algorithms verified on the Tohoku University 6D Pose Estimation Dataset.

Methods	Part 1	Part 2	Part 3	Average
Proposed Method	**96.7%**	**97.3%**	**100**%	**98%**
Original PPF [12]	92%	96.7%	**100**%	96.2%

**Table 8:** Speed of the algorithms verified on the Tohoku University 6D Pose Estimation Dataset (ms/scene).

Methods	Part 1	Part 2	Part 3	Average
Proposed Method	**1982**	**1074**	564	**1207**
Original PPF [12]	2034	1096	**540**	1223

**Table 9:** Pickup success rate for Part A.

Total number of trials	Success	Failure	Success rate
63	**60**	**3**	95.2%

## Supplementary Materials

The following are available online at http://www.mdpi.com/1424-8220/18/8/2719/s1, Video S1: Bin picking experiment.

## References

[1] Holz D., Nieuwenhuisen M., Droeschel D., Stückler J., Berner A., Li J., Klein R., Behnke S., Röhrbein F., Veiga G., Natale C. (2014). Active Recognition and Manipulation for Mobile Robot Bin Picking. The Gearing Up and Accelerating Cross-fertilization between Academic and Industrial Robotics Research in Europe.

[2] Buchholz D. (2015). Introduction–Automation and the Need for Pose Estimation. Bin-Picking: New Approaches for a Classical Problem.

[3] Collet A., Berenson D., Srinivasa S.S., Ferguson D. Object recognition and full pose registration from a single image for robotic manipulation. Proceedings of the 2009 IEEE International Conference on Robotics and Automation (ICRA).

[4] Hinterstoisser S., Cagniart C., Ilic S., Sturm P., Navab N., Fua P., Lepetit V. (2012). Gradient Response Maps for Real-Time Detection of Texture-Less Objects. IEEE Trans. Pattern Anal. Mach. Intell..

[5] Rodrigues J.J., Kim J.S., Furukawa M., Xavier J., Aguiar P., Kanade T. 6D Pose Estimation of Textureless Shiny Objects Using Random Ferns for Bin-picking. Proceedings of the 2012 IEEE/RSJ International Conference on Intelligent Robots and Systems (IROS).

[6] Shroff N., Taguchi Y., Tuzel O., Veeraraghavan A., Ramalingam S., Okuda H. Finding a Needle in a Specular Haystack. Proceedings of the 2011 IEEE International Conference on Robotics and Automation (ICRA).

[7] Liu M.Y., Tuzel O., Veeraraghavan A., Taguchi Y., Marks T.K., Chellappa R. (2012). Fast Object Localization and Pose Estimation in Heavy Clutter for Robotic Bin Picking. Int. J. Robot. Res..

[8] Ulrich M., Wiedemann C., Steger C. (2012). Combining Scale-Space and Similarity-Based Aspect Graphs for Fast 3D Object Recognition. IEEE Trans. Pattern Anal. Mach. Intell..

[9] Rusu R.B., Bradski G., Thibaux R., John H., Willow G. Fast 3D Recognition and Pose Using the Viewpoint Feature Histogram. Proceedings of the 2010 IEEE/RSJ International Conference on Intelligent Robots and Systems (IROS).

[10] Johnson A.E., Hebert M. (1999). Using Spin Images for Efficient Object Recognition in Cluttered 3D Scenes. IEEE Trans. Pattern Anal. Mach. Intell..

[11] Aldoma A., Vincze M., Blodow N., David G., Suat G., Rusu R.B., Bradski G., Garage W. CAD-model Recognition and 6DOF Pose Estimation Using 3D Cues. Proceedings of the 2011 IEEE International Conference on Computer Vision Workshops (ICCV Workshops).

[12] Drost B., Ulrich M., Navab N., Ilic S. Model Globally, Match Locally: Efficient and Robust 3D Object Recognition. Proceedings of the 2010 IEEE Conference on Computer Vision and Pattern Recognition (CVPR).

[13] Wu C.H., Jiang S.Y., Song K.T. CAD-based Pose Estimation for Random Bin-picking of Multiple Objects Using a RGB-D Camera. Proceedings of the 15th International Conference on Control, Automation and Systems (ICCAS).

[14] Mohamad M., Rappaport D., Greenspan M. Generalized 4-points Congruent Sets for 3d Registration. Proceedings of the 2014 International Conference on 3D Vision.

[15] Birdal T., Ilic S. Point Pair Features Based Object Detection and Pose Estimation Revisited. Proceedings of the 2015 International Conference on 3D Vision (3DV).

[16] Wong J.M., Kee V., Le T., Wagner S., Mariottini G.L., Schneider A., Wu J. Segicp: Integrated Deep Semantic Segmentation and Pose Estimation. Proceedings of the 2017 IEEE/RSJ International Conference on Intelligent Robots and Systems (IROS).

[17] Hinterstoisser S., Holzer S., Cagniart C., Ilic S., Konolige K., Navab N., Lepetit V. Multimodal Templates for Real-time Detection of Textureless Objects in Heavily Cluttered Scenes. Proceedings of the 2011 IEEE International Conference on Computer Vision (ICCV).

[18] Katz S., Tal A., Basri R. (2007). Direct Visibility of Point Sets. ACM Trans. Graph..

[19] Rusu R.B., Cousins S. 3D is here: Point Cloud Library (PCL). Proceedings of the 2011 IEEE International Conference on Robotics and automation (ICRA).

[20] Canny J. (1986). A Computational Approach to Edge detection. IEEE Trans. Pattern Anal. Mach. Intell..

[21] Li M., Hashimoto K. (2017). Curve Set Feature-based Robust and Fast Pose Estimation Algorithm. Sensors.

[22] Besl P.J., McKay N.D. (1992). Method for Registration of 3-D Shapes. IEEE Trans. Pattern Anal. Mach. Intell..

[23] Choi C., Taguchi Y., Tuzel O., Liu M.Y. Voting-based Pose Estimation for Fobotic Assembly Using a 3D Sensor. Proceedings of the 2012 IEEE International Conference on Robotics and Automation (ICRA).

[24] Rusu R.B., Blodow N., Marton Z., Soos A., Beetz M. Towards 3D Object Maps for Autonomous Household Robots. Proceedings of the 2007 IEEE/RSJ International Conference on Intelligent Robots and Systems (IROS).

[25] Drost B., Ilic S. 3D Object Detection and Localization Using Multimodal Point Pair Features. Proceedings of the Second International Conference on 3D Imaging, Modeling, Processing, Visualization & Transmission.

[26] Liu D. (2018). Tohoku University 6D Pose Estimation Dataset. https://zenodo.org/badge/latestdoi/143503964.

[27] Drost B., Ulrich M., Bergmann P., Härtinger P., Steger C. Introducing MVTec ITODD — A Dataset for 3D Object Recognition in Industry. Proceedings of the 2017 IEEE International Conference on Computer Vision (ICCV).

[28] Hodan T., Haluza P., Obdržálek Š., Matas J., Lourakis M., Zabulis X. T-LESS: An RGB-D dataset for 6D Pose Estimation of Texture-less Objects. Proceedings of the 2017 IEEE Winter Conference on Applications of Computer Vision (WACV).

